# Low Incidence of Synchronous or Metachronous Tumors after Endoscopic Submucosal Dissection for Early Gastric Cancer with Undifferentiated Histology

**DOI:** 10.1371/journal.pone.0147874

**Published:** 2016-01-25

**Authors:** Chan Hyuk Park, Eun Hye Kim, Jung Hyun Kang, Hyunsoo Chung, Jun Chul Park, Sung Kwan Shin, Sang Kil Lee, Yong Chan Lee

**Affiliations:** 1 Department of Internal Medicine, Guri Hospital, Hanyang University College of Medicine, Guri, Korea; 2 Division of Gastroenterology, Department of Internal Medicine, Severance Hospital, Institute of Gastroenterology, Yonsei University College of Medicine, Seoul, Korea; University of North Carolina School of Medicine, UNITED STATES

## Abstract

**Background:**

Gastric cancer with undifferentiated histology has different clinicopathologic characteristics compared to differentiated type gastric cancer. We aimed to compare the risk of synchronous or metachronous tumors after curative resection of early gastric cancer (EGC) via endoscopic submucosal dissection (ESD), according to the histologic differentiation of the primary lesion.

**Methods:**

Clinicopathological data of patients with initial-onset EGC curatively resected via ESD between January 2007 and November 2014 in a single institution were reviewed. We analyzed the incidence of synchronous or metachronous tumors after ESD with special reference to the differentiation status of the primary lesion.

**Results:**

Of 1,560 patients with EGC who underwent curative resection via ESD, 1,447 had differentiated type cancers, and 113 had undifferentiated type cancers. The cumulative incidence of metachronous or synchronous tumor after ESD was higher in the differentiated cancer group than in the undifferentiated cancer group (*P* = 0.008). Incidence of metachronous or synchronous tumor was 4.8% and 1.2% per person-year in the differentiated and undifferentiated cancer groups, respectively. The Cox proportional hazard model revealed that undifferentiated cancers were associated with a low risk of synchronous or metachronous tumors after adjusting for confounding variables (hazard ratio [95% confidence interval] = 0.287 [0.090–0.918]).

**Conclusions:**

The rate of synchronous or metachronous tumors after curative ESD was significantly lower for undifferentiated cancers compare to differentiated cancers. These findings suggest that ESD should be actively considered as a possible treatment for undifferentiated type EGCs.

## Introduction

Endoscopic submucosal dissection (ESD) is widely used for the treatment of early gastric cancer with expanded indications [1–4]. The expanded indications for ESD, which were proposed by Gotoda *et al*., consist of four categories based on a combination of multiple tumor characteristics including tumor differentiation, depth of tumor invasion, tumor size, presence of ulcers, and presence of lymphovascular invasion [5]. Although the possibility of lymph node metastasis is very low in all four categories [6], Gotoda *et al*. recommended considering surgery for undifferentiated cancers because endoscopic en-bloc resection is sometimes difficult in these cancers [5]. Actually, the curative resection rate is lower for undifferentiated cancers than for differentiated cancers, even for lesions that clearly fulfill the ESD indications [7]. One of the main reasons for the low curative resection rate of ESD for undifferentiated cancers is a higher incomplete resection rate compared to differentiated cancers [1]. Because the margins of undifferentiated early gastric cancers (EGCs) are usually poorly defined, it is difficult to determine the exact lesion size and assess whether the lesion meets ESD indications [8]. However, many studies have revealed that ESD produces good long-term oncologic outcomes for undifferentiated cancers if curative resection has been achieved [7,9,10]. Therefore, ESD can be a good treatment option for selected undifferentiated cancers.

Previously, the clinical and oncologic outcomes after ESD for undifferentiated cancers have been addressed from the perspective of a non-inferiority design compared to outcomes for differentiated cancers. However, we decided to assume that ESD for undifferentiated cancers might be superior to that for differentiated cancers in terms of the risk of synchronous or metachronous tumors.

The undifferentiated type is more frequent than the differentiated type in the *H*. *pylori*-negative gastric cancers [11]. Considering that *H*. *pylori* infection is a well-known risk factor for gastric cancer [12], incidence of synchronous or metachronous tumors might differ between differentiated and undifferentiated type gastric cancers. If the incidence rate of synchronous or metachronous tumors after ESD for undifferentiated cancers would be significantly lower than after ESD for differentiated cancers, then the reason for using ESD for undifferentiated cancers would be strengthened. However, the incidence of synchronous or metachronous tumors after ESD has not been fully evaluated by considering the histologic differentiation of the primary gastric cancer. In this study, therefore, we aimed to compare the risk of synchronous or metachronous tumors after curative resection of EGC via ESD, according to the histologic differentiation of the primary lesion.

## Methods

### Patients

We retrospectively reviewed the clinical data of patients who underwent ESD for initial-onset gastric cancer between January 2007 and November 2014 at Severance Hospital, Seoul, Korea. Patient records/information was anonymized and de-identified prior to analysis. The Institutional Review Board of Severance Hospital approved this study.

Because patients often underwent subsequent surgery after ESD with non-curative resection, we excluded patients with gastric cancer resected non-curatively via ESD for analyzing the risk of synchronous or metachronous tumors. The data were obtained from the prospectively established database of patients who underwent ESD at Severance Hospital, Seoul, Korea. ESD for gastric cancer was performed based on the expanded indication, as previously described [5]: (a) differentiated intramucosal adenocarcinoma less than 3 cm in diameter without lymphovascular invasion, irrespective of the ulcer findings; (b) differentiated intramucosal adenocarcinoma without lymphovascular invasion and negative for ulceration, irrespective of the tumor size; (c) undifferentiated intramucosal cancer less than 2 cm without lymphovascular invasion and ulcer findings; and (d) differentiated adenocarcinoma less than 3 cm with minimal submucosal invasion (<500 μm) and without lymphovascular invasion. When a tumor that fulfilled the expanded indication criteria was removed as a single piece without fragmentation and all lateral and vertical margins were tumor-free on histologic examination, it was considered to have been resected curatively.

Patients were proven to be infected with *H*. *pylori* by the following three methods: (a) histologic evidence of *H*. *pylori*; (b) a positive rapid urease test campylobacter-like organism; (c) a positive ^13^C-urea breath test. *H*. *pylori* infection was defined as a positive result from any of the three tests. In addition, atrophic gastritis and intestinal metaplasia were assessed considering the endoscopic findings [13–16]. The treatment of *H*. *pylori* was decided according to the physician’s recommendation and patient’s preference.

### ESD technique

All ESD procedures were performed with a standard single-channel endoscope (GIF-Q260J or GIF-H260Z, Olympus Optical Co. Ltd., Tokyo, Japan). The typical procedure sequence consisted of marking, mucosal incision, and then submucosal dissection with simultaneous hemostasis. The details of each step are described below.

Firstly, lesion was examined via chromoendoscopy using indigo carmine dye spraying. After making several marking dots circumferentially around the lesion with a needle knife (KD-10Q-1-A, Olympus Optical Co. Ltd., Tokyo, Japan) or a needle knife papillotome (MTW Endoscopy, Wesel, Germany), a saline solution containing epinephrine (0.01 mg/mL) mixed with indigo carmine was injected into the submucosal layer by using a 21-gauge needle in order to lift the lesion away from the muscle layer. A circumferential incision was made in the mucosa by using a needle knife and an insulated-tip knife (KD-610L, Olympus Optical Co. Ltd., Tokyo, Japan). The submucosal layer was dissected directly with various knives until complete removal was achieved. Endoscopic hemostasis was performed with hemoclips or hemostatic forceps whenever bleeding or exposed vessels were observed.

### Histologic evaluation and assessment of resection efficacy

All resected specimens were systematically sectioned at 2 mm intervals, centered on the part of the lesion closest to the margin and the site of the deepest invasion. Histological assessment was based on the Vienna classification [17]. Final pathologic diagnosis was made based on the predominant histology of lesion. Additionally final pathologic diagnoses were classified as gastric cancer with differentiated or undifferentiated histology as Japanese classification [18]. Undifferentiated type included poorly differentiated adenocarcinoma, signet-ring cell carcinoma, and mucinous adenocarcinoma, while differentiated type included papillary adenocarcinoma and well- to moderate differentiated adenocarcinoma.

### Follow-up

All patients underwent an esophagogastroduodenoscopy (EGD) with biopsy, which was scheduled at 3, 6, 12, 18, and 24 months after ESD to check for recurrent tumors. After 24 months, EGD was performed annually. Tumor recurrences found during follow-up EGD were classified into two groups: (a) local recurrence, which was defined as adenoma or cancer detected at the resection site after ESD, (b) adenoma or cancer detected at a gastric site other than the primary resection area after ESD. The latter was further classified into synchronous and metachronous tumor according to the time of recurrence. When adenoma or cancer was detected at a gastric site other than the primary resection area on the follow-up EGD within 12 months after ESD, it was defined as a synchronous tumor. Adenoma or cancer detected at a site other than the primary resection area at 12 months or more after ESD was defined as a metachronous tumor. We analyzed the incidence of recurrence according to the site of recurrence (primary resection site vs. other than primary resection site) and the differentiation status of the primary lesion.

### Statistical analysis

Categorical variables were presented as a sample number with the proportion and were analyzed with the chi-square test or Fisher exact test. Follow-up duration was presented as a median with an interquartile range (IQR), and the data were analyzed with the Mann-Whitney U test. The Kaplan-Meier method and log-rank test were used for the survival analyses. In addition, *P*-value of log-rank test among three groups according to the atrophic gastritis and intestinal metaplasia (IM) was adjusted using the Bonferroni correction. The Cox proportional hazard model was used to adjust for possible confounding variables for age, sex, *H*. *pylori* infection status, atrophic gastritis or IM, tumor differentiation, tumor location, tumor size, presence of ulcer, and depth of tumor invasion. The level of significance was set as *P* < 0.05. All statistical analyses were performed with the statistical software package SPSS for Windows version 18.0 (SPSS Inc., Chicago, IL).

## Results

### Baseline patient and lesion characteristics

A total of 2,118 patients with 2,169 lesions underwent ESD for initial-onset gastric cancer clinically diagnosed as meeting ESD indication. Of them, 558 patients with pathologically non-curative resection were excluded. As a result, the data for 1,560 patients with 1,592 lesions resected curatively via ESD were analyzed. Of these, 1,478 lesions were differentiated cancers and the rest were undifferentiated cancers. Table 1 shows the patient and lesion characteristics according to the histologic differentiation of the tumor.

**Table 1:** Baseline patient and lesion characteristics.

Variable	Primary lesion		*P*-value	Total
	Differentiated cancer	Undifferentiated cancer		
Patients, n	1,447	113		1,560
Lesions, n	1,478	114		1,592
Age, n (%)			<0.001	
≤65 years	806 (55.7)	85 (75.2)		891 (57.1)
>65 years	641 (44.3)	28 (24.8)		669 (42.9)
Sex, n (%)			<0.001	
Male	1,094 (75.6)	61 (54.0)		1,155 (74.0)
Female	353 (24.4)	52 (46.0)		405 (26.0)
*Helicobacter pylori* infection^a^,^b^			0.024	
Absence	226 (21.4)	12 (11.9)		238 (20.6)
Presence	831 (78.6)	89 (88.1)		920 (79.4)
Atrophy and intestinal metaplasia^c^			< 0.001	
None	158 (10.9)	28 (24.8)		186 (11.9)
Atrophic gastritis without IM	391 (27.0)	42 (37.2)		433 (27.8)
Atrophic gastritis with IM	898 (62.1)	43 (38.1)		941 (60.3)
Location, n (%)			0.016	
Upper third	104 (7.0)	9 (7.9)		113 (7.1)
Middle third	271 (18.3)	33 (28.9)		304 (19.1)
Lower third	1,103 (74.6)	72 (63.2)		1,175 (73.8)
Tumor size, n (%)			<0.001	
≤10 mm	703 (47.6)	67 (57.8)		770 (48.4)
10–20 mm	519 (35.1)	47 (41.2)		566 (35.6)
>20 mm	256 (17.3)	0 (0.0)		256 (16.1)
Presence of ulcer, n (%)	69 (4.7)	0 (0.0)	0.008	69 (4.3)
Depth of invasion, n (%)			0.006	
Mucosa	1,385 (93.7)	114 (100.0)		1,499 (94.2)
Submucosa (<500 μm)	93 (6.3)	0 (0.0)		93 (5.8)

^a^*H*. *pylori* infection was tested by using a rapid urease test, a urea breath test, and histologic examination.

^b^Data for *H*. *pylori* infection status were missing in 390 and 12 patients in the differentiated and undifferentiated cancer groups, respectively.

^c^Atrophy and intestinal metaplasia were assessed considering the endoscopic findings.

IM, intestinal metaplasia

Elderly and male patients were more common in the differentiated cancer group compared to the undifferentiated cancer group (differentiated cancer vs. undifferentiated cancer; >65 years, 44.3% vs. 24.8%, *P* < 0.001; male, 75.6% vs. 54.0%, *P* < 0.001). *H*. *pylori* infection was identified in 831 (78.6%) and 89 (88.1%) patients in the differentiated and undifferentiated cancer groups, respectively (*P* = 0.024). Patients with both atrophic gastritis and IM were also more common in the differentiated cancer group than in the undifferentiated cancer group (differentiated cancer vs. undifferentiated cancer; 62.1% vs. 38.1%, *P* < 0.001). Tumor size, presence of ulcer, and depth of tumor invasion did differ between the two groups (*P* < 0.001, *P* = 0.008, and *P* = 0.006, respectively), because all of the undifferentiated cancers that met the expanded indication criteria were less than 2 cm in diameter, without ulcers or lymphovascular invasion.

### Recurrence at the resection site (local recurrence)

**Fig 1:**
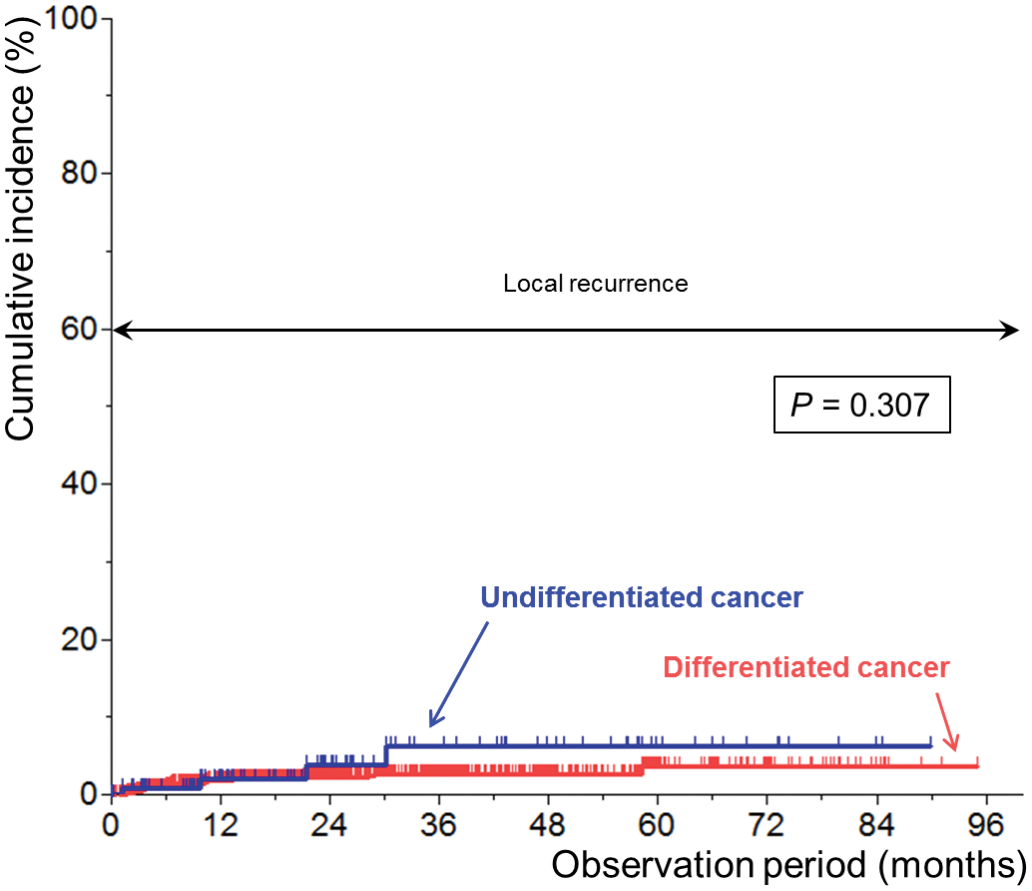
Kaplan-Meier plots for recurrence at the resection site after curative resection according to the histologic differentiation of the primary lesion.

Fig 1 shows the Kaplan-Meier plots for recurrence at the resection site after curative resection according to the histologic differentiation of the primary lesion. The recurrence rate at the resection site within 5 years was 3.6% and 6.2% in the differentiated and undifferentiated cancer groups, respectively. There was no significant difference between the two groups (*P* = 0.307).

### Recurrence at a site other than the primary resection area (synchronous or metachronous tumor)

**Fig 2:**
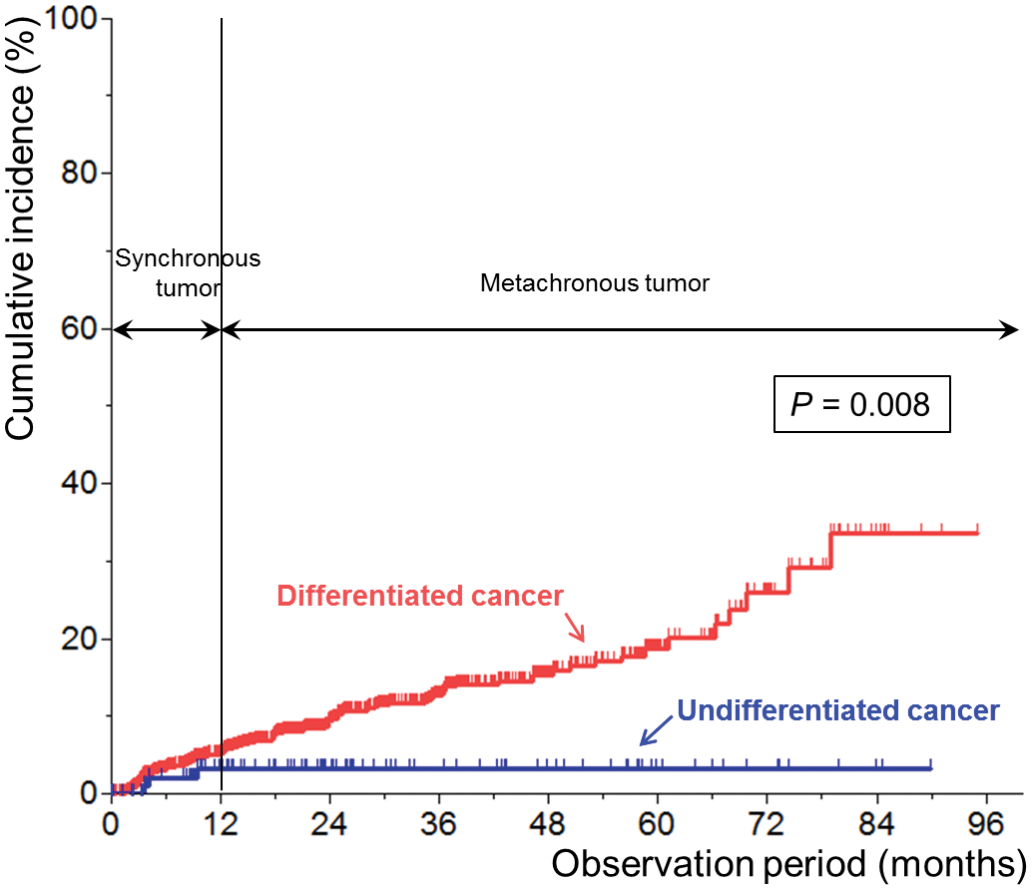
Kaplan-Meier plots for recurrence at a gastric site other than the primary resection site after curative resection according to the histologic differentiation of the primary lesion.

Fig 2 shows the Kaplan-Meier plots for recurrence at a gastric site other than the primary resection area (synchronous or metachronous tumor) after curative resection according to the histologic differentiation of the primary lesion. The median follow-up duration was 16.8 (IQR, 6.5–34.8) months and 22.5 (IQR, 9.3–43.1) months in the differentiated and undifferentiated cancer groups, respectively (*P* = 0.014). Cumulative incidence was significantly higher in the differentiated cancer group than in the undifferentiated cancer group (*P* = 0.008). Incidence of recurrence was 4.8% and 1.2% per person-year in the differentiated and undifferentiated cancer groups, respectively.

For the purpose of exploratory analysis, we compared recurrence rates at a site other than the primary resection area according to the atrophic gastritis and IM status (S1 Fig). The recurrence rate was significantly higher in the atrophic-gastritis-with-IM group than in the no-atrophic-gastritis group (*P* = 0.006). However, the recurrence rate did not differ between the atrophic-gastritis-without-IM group and atrophic-gastritis-with-IM or no-atrophic-gastritis groups (*P* = 0.057 and *P* = 0.558, respectively).

The Cox proportional hazard model was used to adjust for possible confounding variables (Table 2). Undifferentiated histology was significantly associated with a low risk of recurrence at a site other than the primary resection area (hazard ratio [HR] [95% confidence interval [CI]] = 0.287 [0.090–0.918]). Atrophic gastritis with IM was also an associated factor for recurrence at a site other than the primary resection area (with no atrophic gastritis as the reference group: atrophic gastritis without IM, HR [95% CI] = 1.392 [0.653–2.968]; atrophic gastritis with IM, HR [95% CI] = 2.220 [1.098–4.488]). Meanwhile, *H*. *pylori* infection was not associated with recurrence at a site other than the primary resection area (HR [95% CI] = 1.385 [0.751–2.553]). In addition, old age and tumor size were associated factors for recurrence (old age [>65 years], HR [95% CI] = 1.478 [1.043–2.095]; larger tumor size [>20 mm], HR [95% CI] = 0.547 [0.450–2.355]).

**Table 2:** Multivariable analysis for predicting recurrence at a gastric site other than the primary resection area.

Variable	HR (95% CI)	*P*-value
Age		
≤65 years	1	
>65 years	1.478 (1.043–2.095)	0.028
Sex, n (%)		
Male	1.355 (0.864–2.125)	0.185
Female	1	
*Helicobacter pylori* infection^a^		
Absence	1	
Presence	1.385 (0.751–2.553)	0.297
Unknown	1.256 (0.646–2.441)	0.502
Atrophy or intestinal metaplasia^b^		
None	1	
Atrophic gastritis without IM	1.392 (0.653–2.969)	0.392
Atrophic gastritis with IM	2.220 (1.098–4.488)	0.026
Histology, n (%)		
Differentiated	1	
Undifferentiated	0.287 (0.090–0.918)	0.035
Location, n (%)		
Upper third	1.016 (0.487–2.117)	0.967
Middle third	1.438 (0.944–2.191)	0.091
Lower third	1	
Tumor size, n (%)		
≤10 mm	1	
10–20 mm	0.960 (0.662–1.393)	0.831
>20 mm	0.547 (0.301–0.997)	0.049
Presence of ulcer, n (%)	1.030 (0.450–2.355)	0.944
Depth of invasion, n (%)		
Mucosa	1	
Submucosa (<500 μm)	1.128 (0.566–2.250)	0.732

^a^*H*. *pylori* infection was tested by using a rapid urease test, a urea breath test, and histologic examination.

^b^Atrophy and intestinal metaplasia were assessed considering the endoscopic findings.

HR, hazard ratio; IM, intestinal metaplasia

### Clinicopathological characteristics of recurrent tumor at a site other than the primary resection area

The detailed clinicopathological characteristics of recurrent tumor at a gastric site other than the primary resection site are shown in Table 3. During the follow-up period, 128 tumors were identified as synchronous or metachronous tumors in the differentiated cancer group. About half of the tumors were synchronous tumors and the others were metachronous tumors. In contrast, only three synchronous tumors were identified in the undifferentiated cancer group. There were no metachronous tumors in the undifferentiated cancer group. In the differentiated cancer group, 6.3% of synchronous or metachronous tumors were identified as undifferentiated cancers. In the undifferentiated cancer group, all three synchronous tumors developed as adenoma or differentiated cancers.

**Table 3:** Clinicopathological characteristics of recurrent tumor at a gastric site other than the primary resection area according to the histologic differentiation of the primary lesion.

Variable	Primary lesion		*P*-value
	Differentiated cancer	Undifferentiated cancer	
Patient, n	128	3	
Type of recurrence, n (%)			0.119
Synchronous tumor	62 (48.4)	3 (100.0)	
Metachronous tumor	66 (51.6)	0 (0.0)	
Histology, n (%)			0.386
Adenoma	81 (63.3)	1 (33.3)	
Differentiated cancer	39 (30.5)	2 (66.7)	
Undifferentiated cancer	8 (6.3)	0 (0.0)	
Location, n (%)			>0.999
Upper third	22 (17.2)	0 (0.0)	
Middle third	31 (24.2)	1 (33.3)	
Lower third	75 (58.6)	2 (66.7)	
Tumor size, n (%)			0.675
≤10 mm	80 (62.5)	3 (100.0)	
10–20 mm	35 (27.3)	0 (0.0)	
>20 mm	13 (10.2)	0 (0.0)	

### Additional treatment for recurrent tumor at a site other than the primary resection area

Additional treatments for recurrent tumor at a site other than the primary resection site are described in Table 4. More additional treatments were required in the differentiated cancer group than in the undifferentiated cancer group (7.8% vs. 1.8%, *P* = 0.018). Of the three synchronous tumors in the undifferentiated cancer group, two (that were differentiated cancers) were treated with endoscopic resection. The remaining tumor, an adenoma, was closely observed.

**Table 4:** Additional treatment for recurrent tumor at a gastric site other than the primary resection area.

Variable	Primary lesion		*P*-value
	Differentiated cancer	Undifferentiated cancer	
Patient, n	1,447	113	
Synchronous or metachronous tumor, n	128	3	
Additional treatment for synchronous or metachronous tumor, n (%)^a^	113 (7.8)	2 (1.8)	0.018
Surgery	12 (0.8)	0 (0.0)	>0.999
Endoscopic resection	89 (6.2)	2 (1.8)	0.056
APC ablation	11 (0.8)	0 (0.0)	>0.999
Palliative chemotherapy	1 (0.1)	0 (0.0)	>0.999
Observation for synchronous or metachronous lesion, n (%)	15 (1.0)	1 (0.9)	>0.999

^a^The proportion of variables was calculated from the number of enrolled patients.

APC, argon plasma coagulation

## Discussion

Metachronous tumor after surgery for gastric cancer has been considered to be of lesser importance compared to local recurrence or extra-gastric recurrence because recurrence in a remnant stomach is rare [19–21]. In the era of ESD, however, metachronous tumors can no longer be ignored. Because the ESD method preserves the whole stomach, the incidence of metachronous tumors after ESD is higher than after surgery [20–22]. Risk of metachronous tumors is therefore a concern even if the primary lesion is treated successfully [21,23]. Almost all recurrent tumors require additional treatments, and these will be a burden for patients who undergo ESD [21].

In our study, we showed that the local disease control rate after curative resection via ESD was excellent regardless of the degree of differentiation of the primary lesion. However, patterns of recurrence at a gastric site other than the primary resection site did differ according to the histologic differentiation of the primary lesion. The incidence of synchronous or metachronous tumors after ESD for differentiated cancers remained steady over time, with an annual incidence of 3.7%. At 5 years after ESD, synchronous or metachronous tumors developed in about 19% of patients who had undergone curative resection via ESD. More metachronous tumors would be expected to develop over a longer follow-up. In contrast to the results of ESD for differentiated cancers, the incidence of synchronous or metachronous tumors after ESD for undifferentiated cancers was relatively low. Within 5 years after ESD, only 3% of patients showed recurrence at a site other than the primary resection site. Moreover, no metachronous tumor was identified during the follow-up period. These findings suggest that ESD for undifferentiated cancers may have better outcomes compared to that for differentiated cancers. If curative resection is achieved via ESD, the long-term oncologic outcome of undifferentiated cancers may be superior to that of differentiated cancers in terms of the incidence of synchronous or metachronous tumors after ESD.

The different patterns of recurrence after ESD may be due to the different pathogenesis of gastric cancer according to the histologic differentiation. It has long been known that the development of differentiated cancers is associated with *H*. *pylori* infection. Iseki *et al*. demonstrated that differentiated adenocarcinomas were usually located in areas of mucosa infected with *H*. *pylori*, while undifferentiated adenocarcinomas were frequently located in non-infected areas [12]. Chronic gastritis due to *H*. *pylori* infection may induce intestinal-type gastric cancer via atrophic gastritis, IM, and dysplasia [24–27]. Because atrophic gastritis and IM can spread extensively due to persistent inflammation, additional dysplasia or gastric cancer may occur over time in patients with differentiated cancers. On the other hand, the rest of the gastric mucosa may be relatively normal in patients with undifferentiated cancers. Therefore, the risk of metachronous tumor may be relatively low in these patients. Our finding that atrophic gastritis with IM was an associated risk factor for development of synchronous or metachronous tumor also supports a hypothesis for pathogenesis of gastric cancer known as Correa’s postulation.

In contrast to the results of atrophic gastritis and IM, *H*. *pylori* infection status did not have any apparent influence on the development of synchronous or metachronous tumor after ESD in our study. In addition, *H*. *pylori* infection was more common in the undifferentiated cancer group than in the differentiated cancer group, while atrophic gastritis with IM was more commonly identified in the differentiated cancer group. These results may support that *H*. *pylori* does not survive well in atrophic or IM mucosa [28]. Because patients with negative results for *H*. *pylori* infection include both *H*. *pylori* never-infected and past-infected patients, histologic differentiation of primary EGC and atrophic gastritis with IM, rather than *H*. *pylori infection* status, may be more useful for predicting development of synchronous or metachronous tumor after ESD.

Another potential benefit of ESD for undifferentiated cancers is that a relatively longer gastrectomy-free survival may be expected for patients with undifferentiated cancers, because undifferentiated cancers are more common in a younger population than are differentiated cancers [29]. When considering the quality-adjusted life years of patients with gastric cancer after ESD or surgery, ESD should be seriously considered as a first-line treatment in young patients, especially in those with undifferentiated gastric cancers.

However, the low complete and curative resection rate is still a matter of concern in ESD for undifferentiated cancers [1,7]. Despite various efforts to delineate the margin of undifferentiated cancers, it is still difficult to discern the exact margin via endoscopy. Although magnifying endoscopy with narrow-band imaging helps in the successful delineation of gastric cancers that show an unclear margin, its usefulness for undifferentiated cancers has not been proven [8]. One attempt to overcome this limitation is an assessment of the margins by using confocal laser endomicroscopy (CLE). CLE allows real-time, *in vivo* high-resolution and high-magnification imaging of the gastrointestinal epithelium, which is comparable in accuracy to histopathology [30]. Previous studies showed that CLE could provide an accurate diagnosis for gastric cancer and adenoma [30,31]. Although there is currently a lack of evidence that CLE is useful to delineate the cancer margin, a prospective study on delineation of the margin of EGC by using CLE is ongoing (ClinicalTrials.gov Identifier: NCT02189226).

Although this study was the first to evaluate the incidence of recurrence at a gastric site other than the primary resection site according to the histologic differentiation of the primary lesion, the results should be interpreted cautiously because our study has several limitations. The first limitation is the retrospective design of the study; however, all of the data were collected prospectively for a future analysis of ESD performance. Accordingly, this limitation introduces only a slight chance for bias. The second limitation is that lesion characteristics did differ between the differentiated and undifferentiated groups because the expanded indication criteria for ESD are different depending on the histologic differentiation of EGC. Differentiated cancers that fulfill the expanded indication criteria for ESD can include larger tumors (>3 cm) and tumors with ulcer or minute submucosal invasion (<500 μm), unlike undifferentiated cancers. Therefore, we cannot conclude for certain that undifferentiated cancers always have a low incidence rate of synchronous or metachronous tumor compared to any other subgroups of differentiated cancers. Even with the limitations of our study, we can conclude that undifferentiated cancers that meet the expanded indication criteria have a lower incidence rate of synchronous or metachronous tumors than differentiated cancers that fulfilled the expanded indication criteria. The exclusion of non-curatively resected lesion is the third limitation of the study. Because patients underwent surgery when the primary lesion had been resected non-curatively, we could not include them for analysis of metachronous tumor. Therefore, we can say that the incidence rate of synchronous or metachronous tumors may be lower after ESD for undifferentiated cancers than after ESD for differentiated cancers only if curative resection has been achieved. The fourth limitation is a relatively short follow-up duration especially in the differentiated cancer group. Although bias due to the censored data can be a concern for interpreting survival data, however, it may be adjusted by the Kaplan-Meier method and the Cox proportional hazard model. In addition, we think that relatively short follow-up duration in the differentiated cancer group would not be a big problem, because the gap of incidence of metachronous tumor between the differentiated and undifferentiated cancer groups may expand if the follow-up duration in the differentiated cancer group increases. Finally, the fifth limitation is that atrophic gastritis and IM, which are risk factors for gastric cancer, were evaluated based on gross endoscopic findings only. Atrophic gastritis and IM can be assessed more precisely by operative link on gastritis assessment (OLGA) and operative link on gastric IM assessment (OLGIM) staging, respectively [32,33]. OLGA and OLGIM, however, require at least five random biopsies. As these assessment methods are rather impractical, we decided to use the assessment of atrophic gastritis and IM based on gross endoscopic findings.

Despite these limitations, our data may form the basis of a system to understand the benefit of ESD for undifferentiated cancers. The incidence rate of recurrence at a gastric site other than the primary resection site after curative resection was significantly lower after ESD for undifferentiated cancers than after ESD for differentiated cancers. ESD for undifferentiated cancers can be actively considered as a beneficial treatment when the lesion would be expected to be resected curatively.

## Supporting Information

S1 Fig Kaplan-Meier plots for synchronous or metachronous tumor after curative resection according to the atrophic gastritis and intestinal metaplasia status. IM, intestinal metaplasia.
(TIF)
