# Supplementary figures and images for: Low Incidence of Synchronous or Metachronous Tumors after Endoscopic Submucosal Dissection for Early Gastric Cancer with Undifferentiated Histology
Source: PLoS One. 2016 Jan 25;11(1):e0147874. doi: 10.1371/journal.pone.0147874 (PMC4726500; doi:10.1371/journal.pone.0147874)

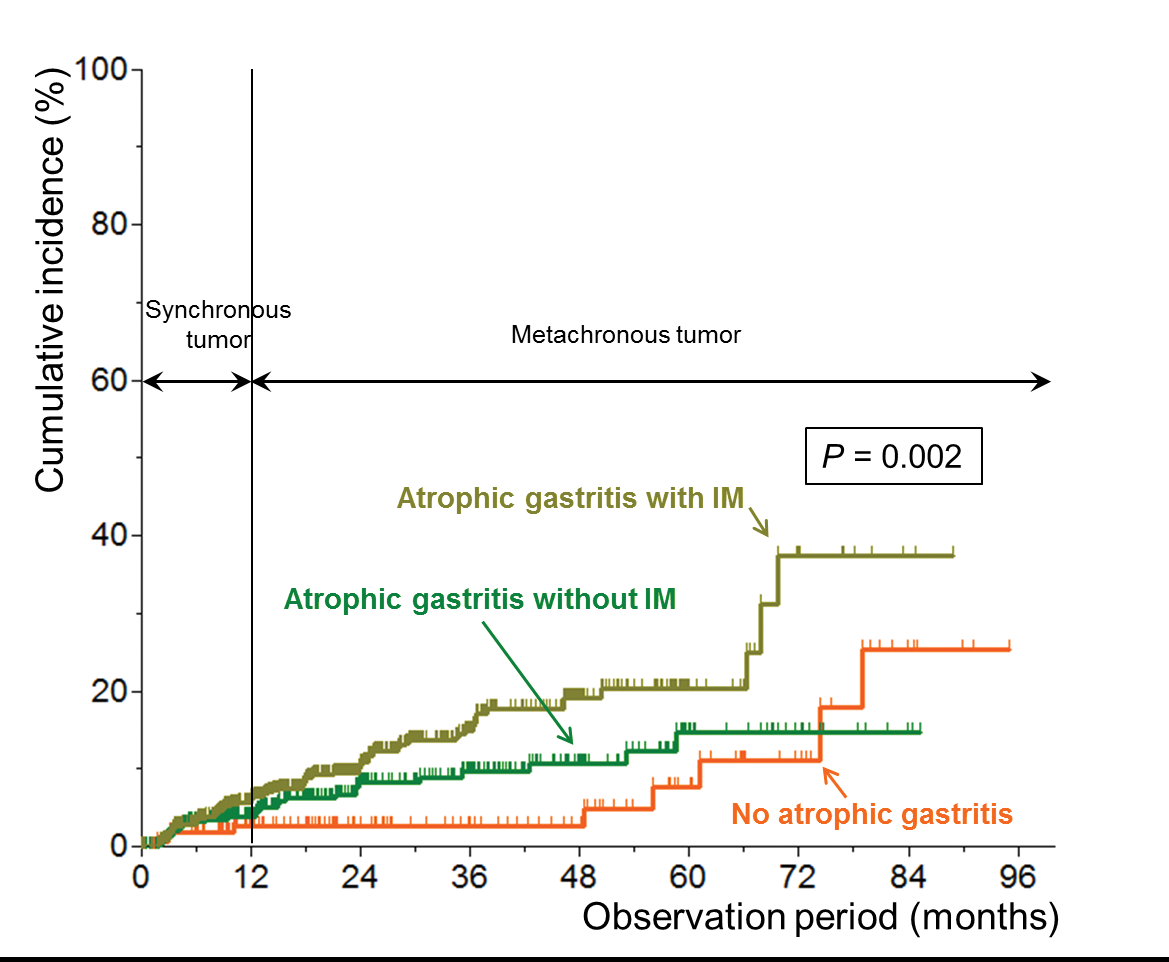

Supplement: S1 Fig — IM, intestinal metaplasia. (TIF) [file pone.0147874.s001.tif]
